# Promoting equity in adolescent health in Latin America: designing a comprehensive Sex education program using Intervention Mapping. A mixed methods study

**DOI:** 10.3389/frph.2024.1447016

**Published:** 2024-11-18

**Authors:** Betzabé Torres-Cortés, Loreto Leiva, Katia M. Canenguez, Lorraine Greaves

**Affiliations:** ^1^ Department of Psychology, Faculty of Social Sciences, Pontificia Universidad Católica de Chile, Santiago, Chile; ^2^ Department of Psychology, Faculty of Social Sciences, Universidad de Chile, Santiago, Chile; ^3^ Millennium Institute for Research on Depression and Personality (MIDAP), Santiago, Chile; ^4^ Department of Psychiatry, Harvard Medical School, Boston, MA, United States; ^5^ Department of Psychiatry, Massachusetts General Hospital, Boston, MA, United States; ^6^ Centre of Excellence for Women’s Health, Vancouver, BC, Canada; ^7^ Faculty of Medicine, School of Population and Public Health, University of British Columbia, Vancouver, BC, Canada

**Keywords:** sex education, adolescents, health equity, gender equity in health, Intervention Mapping, framework for gender-transformative health promotion, gender equity

## Abstract

**Introduction:**

Implementing sex education programs during adolescence is crucial for addressing the risks associated with sexuality. However, some of these interventions lack proper incorporation of a gender perspective and maintain a heteronormative and biologically-focused approach, potentially resulting in inequitable outcomes for adolescents. In response, comprehensive sex education is most effective due to its multidimensional view of sexuality. However, integrating a comprehensive perspective on sexuality and a gender lens that contributes to adolescent health equity presents challenges, especially in low and middle-income countries. This study aimed to develop a comprehensive and gender-transformative sex education program for adolescents in a middle-income country of Latin America, utilizing the Intervention Mapping Approach.

**Methods:**

This exploratory sequential mixed-method study comprised two phases. In the first phase, a literature review, nine focus groups with high school students, and 14 interviews with school professionals were conducted to inform program design. Subsequently, the program underwent validation through expert judgment. In the second phase—as part of program development—a preliminary evaluation was conducted by implementing the program in two high schools with 30 students from public high schools, who were administered a pre-post *ad hoc* survey.

**Results:**

A comprehensive and gender-transformative sex education program was designed based on literature review findings and input from students and school workers. The *ad hoc* survey revealed a statistically significant increase in protective skills in sexuality (*W* = 59, *p* = .01) among all participants.

**Discussion:**

Designing a sex education intervention through Intervention Mapping allowed for the integration of evidence and the needs of the target population. The results of the preliminary evaluation suggest the potential of the developed program to enhance protective skills in sexuality and promote health equity through gender-equitable outcomes in adolescent sex education.

## 1 Introduction

Adolescence marks a period where sexuality is a normative aspect of development (1). However, adolescents encounter specific challenges, such as physical, psychological, and sexual changes, along with the exploration of sexual orientation and gender identity. Insufficient access to information and difficulties in assessing risk further compound their vulnerability in sexual experiences (2–5). These challenges can manifest in risky sexual behavior (6), predisposing adolescents to sexually transmitted infections (STIs), unintended pregnancies, and interpersonal or legal conflicts (7). Moreover, adverse sexual experiences are associated with detrimental mental health outcomes including depression, negative perceptions of sexual relationships, anxiety, substance use, post-traumatic stress disorder, and suicidal ideation/suicidality (8–12). Notably, these complexities are exacerbated in contexts where adolescents encounter additional adversities such as sexual violence, limited educational opportunities, elevated prevalence of STIs, economic disparities, gender-based discrimination, heightened vulnerability stemming from ethnic backgrounds, and barriers to accessing sexuality related information, factors that are particularly pronounced in low and middle-income countries (13–18).

Sexuality represents a complex facet of human identity, influenced by a myriad of factors such as biology, psychology, culture, religion, and spirituality (19). Gender identity, and gender roles, norms, stereotypes, attributes, and behaviors deemed suitable by society based on an individual's gender, intricately shapes self-perception, interactions, and power dynamics. In this way, gender closely intertwines with sexuality as social norms, partly constructed by gender roles, influence the experience of sexuality (20–22). During adolescence, gender disparities become evident in the impact of sexual experiences. Early initiation of sexual activity may lead to a decline in well-being, particularly impacting females (23, 24). Non-binary adolescents may encounter internalizing or externalizing symptoms stemming from inadequate support, social exclusion, and bullying (25–27). Additionally, partner violence has been linked with mental health challenges in females (28, 29), highlighting a significant correlation. Furthermore, sexual health services predominantly cater to girls, often neglecting consideration for boys (30, 31).

The preceding points emphasize the urgent need for preventive actions, primarily implemented through sex education programs [SEPs]. In many countries, especially in Latin America, these interventions predominantly occur within educational institutions (32) and are marked by an emphasis on oversimplified biological perspectives and traditional gender norms (32–34). However, ongoing efforts are underway to transition sex education towards mitigating inequalities by embracing a more inclusive approach to sexuality (32). In Chile, a law enacted in 2010 mandated sex education within public schools and high schools (35). Consequently, schools are required to deliver sex education either by contracting a program from an external institution or by developing their own. Most of these interventions are internally developed by the school or the local authority (municipality), with only a small percentage of schools implementing an external program (36). Within these SEPs, a predominant risk-based approach is prevalent (37), prioritizing biological aspects, often to the detriment of other considerations (36).

In addition to this risk-based approach, there are two more perspectives in sexual education. The abstinence approach promotes delaying sexual activity until marriage, presenting it as the most effective way to prevent pregnancy and STIs (38, 39). Within this approach is abstinence-plus, which also teaches about sexual healthcare methods (40). On the other hand, the comprehensive approach encompasses various interventions that share common features such as the incorporation of abstinence and sexual self-care methods, a human rights and gender perspective, and coverage of biological, psychosocial, spiritual, and value-based topics (39, 41–49).

Some studies have shown that abstinence-plus SEPs can increase knowledge about sexuality, reduce the risk of STIs, and enhance self-efficacy (40, 50). However, meta-analyses, systematic reviews, and other studies have demonstrated that abstinence-focused interventions -compared to comprehensive interventions- are largely ineffective, as they have limited success in promoting abstinence and preventing pregnancies (38, 47, 51–53). Additionally, these programs fail to meet the needs of sexually active adolescents, reinforce gender stereotypes, and contribute to stigma and mental health challenges for lesbian, gay, bisexual, and transgender [LGBT] youth (54, 55).

In contrast, evidence indicates that more effective SEPs are characterized by a comprehensive approach, as they not only augment knowledge about sexual health but also demonstrate effectiveness in reducing rates of STIs and unintended pregnancies, delaying the onset of sexual intercourse, and positively impacting mental health (16, 38, 47, 48, 56–59). Furthermore, they can help prevent partner violence, reduce homophobia, improve access to sexual health information, and increase confidence in discussing sexual topics (58, 60–62).

However, despite these positive outcomes, further research is essential to evaluate the impact of comprehensive interventions on vulnerable groups and the integration of gender-related elements (59, 63, 64). Additionally, in middle- and low-income countries, significant gaps persist in the development and implementation of SEPs, accompanied by a scarcity of experimental research (32, 64–66).

The importance of gender within sex education is highlighted in contexts where gender inequality is deeply ingrained (67). Outcomes of such interventions may detrimentally impact the sexual and mental health of adolescents (68), contributing to gender inequity in health. Certain programs may disproportionately benefit heterosexual adolescents over their LGBT counterparts (69, 70); and some programs inadvertently reinforce gender stereotypes of boys and girls and/or foster stigma in LGBT adolescents, leading to mental health difficulties (54, 55, 71). This issue is pronounced in numerous countries, including in Latin America (33). Therefore, there is a need for SEPs that promote gender equity, which is achieved through interventions that actively challenge detrimental gender norms, promoting more balanced gender relations (72) as everyone, regardless of gender, has the right to equal access to health, well-being, and structures providing health services (73).

In this context, design guidelines for SEPs recommend adopting a gender-transformative approach that emphasizes empowerment and gender equity in relationships (74). The Framework for Gender-Transformative Health Promotion [FGTHP] promotes this, asserting that interventions should prioritize transforming gender roles, relations, and practices, aiming for both health-improving and transformative outcomes to address harmful gender norms (75, 76). This framework facilities the exploration of health promotion strategies grounded in gender ideologies, discriminatory behaviors, and avenues for improving public health, while reshaping gender norms, roles, and relations through the intervention (77). Within this study, the FGTHP will guide the examination of evidence regarding individuals, context, and the issue through a gender lens.

On the other hand, designing health-promoting SEPs requires evidence-based, locally tailored approaches (64, 78). Intervention Mapping [IM] is a suitable framework, offering an ecological-systemic perspective, evidence-based foundations, and a participatory methodology to align with the needs and context of the target population (79). The effectiveness of IM is evident in its application to various sex education interventions (80–83) following some or all of its 6-step protocol (84). These steps involve developing Logic Models for Problem and for Change, selecting theory-based methods, crafting an Intervention Logic Model, determining program structure and content, creating an implementation plan, and establishing an evaluation plan to assess program outcomes.

In summary, this background underscores the need for evidence-based preventive interventions focused on sexual health in adolescence, with a proven efficacy of a comprehensive sexual education approach. Additionally, it is recognized that addressing gender within SEPs is crucial for achieving equity in health, particularly given the unique challenges faced by low and middle-income countries, where sex education often follows traditional gender perspectives and lacks comprehensive interventions. Hence, the aim of this research is to design a comprehensive, gender-transformative sex education program to contribute to health equity in adolescents. This will be achieved by implementing the first four steps of IM in conjunction with the Framework for Gender- FGTHP as key methodologies.

## 2 Method

This study employs an exploratory sequential mixed-method design, starting with qualitative data collection followed by quantitative assessment (85). The variant “intervention design” was utilized, where qualitative insights guided the development of a meaningful program for participants, subsequently evaluated quantitatively (85).

**Figure 1:**
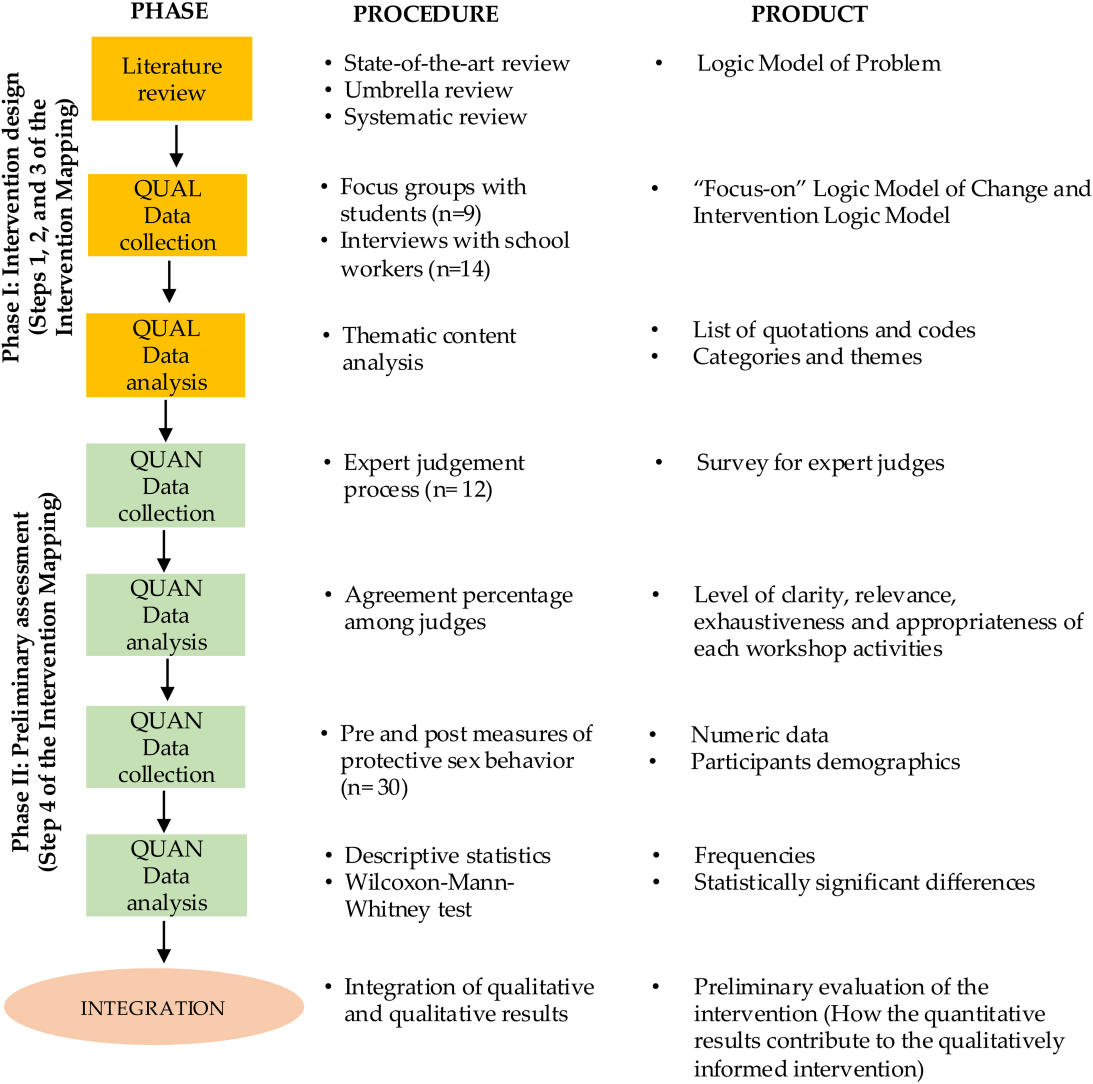
Design of the study (85).

This process unfolded in two phases (see Figure 1): the first involved a literature review and qualitative data collection for intervention design through focus groups and interviews. The second phase encompassed validation of the intervention design (*via* expert judgment) and a preliminary evaluation. Throughout these phases, steps one to four of the IM framework were implemented.

### 2.1 Phase I: intervention design

#### 2.1.1 Participants

A typical case sampling method was employed (86), selecting participants based on their potential to provide information on SEPs.

To choose participants, six public high schools were selected in collaboration with the Santiago Municipality's Department of Education, which oversees these institutions. The sample included three co-educational high schools, one single-sex boys' high school, and two single-sex girls' schools. These schools serve students from middle- and low-income backgrounds, many of whom come not only from Santiago but also from nearby cities. Two of these high schools offer technical training in the last two years of study, while the others provide a liberal arts and sciences education. Additionally, there is a significant percentage of students from other countries.

The inclusion criteria for professionals were as follows: they had to be responsible for the SEP at their school or have implemented sex education activities within the past year. All professionals in charge of the SEP at their school were invited to participate, and everyone accepted (*n* = 6). One teacher from each school was also invited to be part of the study. All accepted to participate, and two additional teachers who showed interest were included as well (*n* = 8). Thus, 14 school professionals were involved in this phase.

For students, the inclusion criteria specified that they must be first- or second-year high school students who participated in sexual education activities in the past year and have obtained informed consent from their parents. A general invitation to participate in the study was extended to all first- and second-year high school students at the participating schools, resulting in 56 students agreeing to take part, representing a range of sexual orientations and gender identities.

• The distribution of students in each focus group was based on their available schedules, resulting in different numbers and compositions of participants for each group. There was no separation by gender in the coeducational high schools, as the research team prioritized grouping students according to their scheduling preferences. The composition of each focus group was as follows:
• Single-sex girls' schools: (i) nine female students, (ii) seven female students, (iii) four female students, (iv) six female students
• Single-sex boys' high school: (v) four male students, (vi) six male students, and one student who identified as a transgender woman
• Co-educational high schools: (vii) seven students (two male and five female), (viii) six students (three male and three female), (ix) six students (four male and two female)

#### 2.1.2 Data collection techniques

Nine focus groups with students and 14 interviews with professionals were conducted. The dimensions addressed in both techniques included sexuality needs for adolescents, components of a school-based sexual intervention, methodological considerations, and relevant topics. Examples of the interview and focus group guide can be found in Supplementary Material 1.

#### 2.1.3 Analysis

A content analysis was conducted using categories derived from empirical data (87). To accomplish this, the thematic content analysis procedure proposed by Díaz Herrera (88) was adhered to, entailing category configuration, development of category trees, category validation, and identification of central themes.

#### 2.1.4 Procedure

During Phase I, steps one, two, three, and part of four of the IM were implemented. In step one, a needs assessment was carried out to construct a Logic Model of the Problem. This assessment started with a state-of-the-art review and an umbrella review, focusing on adolescent sexuality, SEPs, and topics pertinent to comprehensive interventions. Health equity aspects, particularly those related to gender equity, were emphasized in the search. Then, information from a systematic review previously conducted by the team (documented in a separate publication) was incorporated. The obtained information informed the Logic Model of the Problem and the script for interviews and focus groups.

Subsequently, professionals signed informed consent forms. Parents provided consent through one of the following methods: (i) during parent meetings at the school, where the research team explained the study, or (ii) individually, with the teacher explaining the study to the parents. During this process, approximately 10% of parents did not authorize their children's participation due to religious beliefs or disagreements with the gender approach. Afterward, the team visited students at the high schools to explain the study and obtain their assent.

Next, interviews and focus groups were conducted, and the information underwent content analysis. By integrating insights from the literature review, focus groups, and interviews the problem was delineated, and its personal and environmental determinants were identified, leading to the development of the Logic Model of the Problem.

Step two involved developing the Logic Model of Change, outlining program outcomes, and examining theoretical models identified through the systematic review. In this process, the personal and contextual factors (identified in the Logic Model of the Problem) that could be addressed through the intervention were selected.

Finally, step three involved selecting theoretically and empirically based methods, practical applications, and intervention components through a systematic review, in conjunction with results from qualitative techniques. This informed the Intervention Logic Model, specifying program structure, topics, workshop sessions, and activities. Additionally, performance objectives were developed to address the personal and contextual determinants.

### 2.2 Phase II: validation and preliminary evaluation of the intervention

#### 2.2.1 Participants

The expert judgment involved 12 professionals who met specific criteria, including academic backgrounds in Education or Psychology, experience within educational communities, and previous involvement in sex education interventions or research. The professional cohort consisted of Teachers, Psychiatrists, Social Workers, a Stakeholder, Nurse-Midwives, Psychologists, and an Educational Psychologist.

In the preliminary evaluation a non-probabilistic sampling approach was utilized. The student cohort comprised 30 first- and second-year students from two public high schools in Santiago, Chile, averaging 15.1 years of age (SD = .923). Of these, 66.7% were Chilean, with 53.3% not affiliating with any religious belief and 29.9% identifying with a religion (Catholic, Protestant, or Mormon). In terms of gender identity, 50% identified as women, 40% as men, and 10% specified other identities (e.g., gender fluid, he-she). Regarding sexual orientation, 66.7% identified as heterosexual, 13.3% were undefined, 10% as bisexual, and 10% indicated other orientations (e.g., pansexual, aromantic). Participants were presented with various gender identity and sexual orientation options, with reported percentages reflecting adolescents' selections.

#### 2.2.2 Measures and data collection

An intervention validation survey was developed for expert judgment, comprising four dimensions evaluated dichotomously (Yes-No) for each session activity: (i) Precision—clarity for effective implementation by facilitators; (ii) Relevance—contribution to achieving objectives; (iii) Comprehensiveness—inclusion of sufficient elements aligned with theoretical foundations; (iv) Adequacy—appropriateness for the participants. Experts provided qualitative feedback, including suggestions for improvement.

For the preliminary evaluation, an ad-hoc survey measured protective skills in sexuality, defined for this study as actions or intentions aimed at preventing STI and unintended pregnancies, while enhancing factors associated with sexuality-related empowerment. The survey utilized seven Likert-scale items (from strongly disagree to strongly agree), where a higher score indicated better protective skills in sexuality. This scale was derived from the literature and instruments used in similar studies sharing the same intervention theoretical foundation, as seen in works by Constantine et al. (57), Coyle et al. (89), and Manaseri et al. (90).

#### 2.2.3 Data analysis

The expert judgment results were evaluated based on the percentage of agreement among judges for each activity across sessions. Activities with less than 70% agreement were adjusted according to judges' guidelines.

The survey's reliability was assessed using McDonald's Omega coefficient, which is suitable for ordinal-level variables due to its sensitivity and unbiased estimation (91–93). Scale interpretation considered values above.90 as excellent,.80 as good, and.70 as acceptable (94). Data distribution was evaluated using the Shapiro-Wilk test (95). Subsequently, the Wilcoxon–Mann–Whitney test (96, 97) compared pre- and post-intervention protective skills in sexuality across 30 paired observations, with significance determined at the.01 level to check whether the median difference between the pre and post was statistically significant. All analyses were performed using Jamovi version 2.3.18.0.

#### 2.2.4 Procedure

During this phase, part of step four of the IM was executed (production and pre-testing of the program). The expert judgment process was conducted online. An email was sent with details about the process and program sessions relevant to each expert's area of expertise, ensuring a minimum of three reviewers per session. Experts were then asked to complete an intervention validation survey via an online platform, accompanied by an instructional video explaining the program design process and evaluation instructions. Adjustments to the sessions were implemented based on the recommendations provided by the expert judges.

Following this, a preliminary program evaluation was conducted by implementing it in a small sample from the target population across two high schools within the demographic. Parents provided informed consent, followed by assent from students who completed an entry survey. Workshops were facilitated by two teachers in each high school—two Mathematics teachers, one English language teacher, and Biology teacher—following training from the research team. The 10 sessions occurred weekly during class time. Upon completion of the intervention, an exit survey was administered to the students.

### 2.3 Ethical considerations

The study was conducted with the authorization of the Universidad de Chile Social Sciences Ethics Committee, which is accredited by the Regional Ministry of Health. Participation was voluntary and confidential. Expert judges and parents provided signed informed consent forms, while students provided signed assent forms. The characteristics of the study and the assurance of participants' rights were ensured and were explicitly outlined in the consent and assent forms.

## 3 Results

### 3.1 Phase I: intervention design

#### 3.1.1 Literature review

The comprehensive analysis, combining the state-of-the-art and umbrella reviews, identified key personal and environmental factors influencing the intervention's main concerns: sexual risk behavior, gender inequity in sexual health, and mental health issues linked to sexuality. The literature review underscored key elements such as behaviors conducive to positive adolescent sexual health, contributors to favorable mental health outcomes related to adolescent sexuality, and promoters of gender equity in adolescent sexual health. Detailed citations for these studies are provided in Supplementary Material 2.

These results facilitated the creation of the Logic Model of the Problem (Supplementary Material 3). This model provides a detailed depiction of the concerns addressed in the intervention by emphasizing the individual determinants involved. These determinants encompass factors such as knowledge, attitudes, risk perception, or skills that are correlated with or could potentially impact adolescent risky sexual behavior (98). Additionally, it outlines specific behaviors linked to this issue and their repercussions on adolescent health.

Additionally, the results of the systematic review (99), provide information for the subsequent design of the Logic Model of Change. These results highlighted that the majority of SEPs were grounded on behavioral change theories, primarily Ajzen's Theory of Planned Behavior (100) and Ajzen & Fishbein's Theory of Reasoned Action (101). Additionally, a substantial percentage of interventions drew from two or more theoretical frameworks. Key insights from this review include the prevalence of comprehensive approaches, participatory methodologies, and the importance of facilitator training. While no standardized dosage was identified, most interventions entailed a minimum of 10 h of weekly delivery.

#### 3.1.2 Focus groups and interviews

The content analysis identified key themes across three dimensions, outlined in Table 1. Within dimension one “Components of a sexual intervention for adolescents,” emerged themes included: (i) specific facilitator guidance, (ii) extended intervention time, and (iii) facilitator training. Dimension two “Relevant topics for the intervention” encompassed: (i) STI and pregnancy prevention methods, (ii) gender dynamics, (iii) LGBT issues, (iv) intimate partner violence, and (v) emotional aspects. Finally, dimension three “Methodological considerations” highlighted: (i) the implementation of participatory strategies and (ii) utilization of dynamic activities. In summary, the findings underscored the importance of facilitator training, structured interventions, participatory methodologies, tailored content for LGBT students, integration of gender equity principles, and adaptation of a multidimensional approach to sexuality education.

**Table 1:** Central themes identified in focus groups and interviews.

Dimension	Central themes	Description	Quotes
Components of a sex education intervention for adolescents	Specific guidelines for facilitators Increased intervention time Training for facilitators	School professionals underscore the necessity of an intervention handbook encompassing activities for each session. This would alleviate their workload, given their typically overwhelming responsibilities. Moreover, it would facilitate standardization in curriculum delivery, irrespective of the personal beliefs or values of facilitators, who are predominantly teachers in most high schools. Both professionals and students identify multiple topics necessitating inclusion, highlighting the need for an extended intervention to adequately address these subjects. Not all professionals are knowledgeable in the subject matter; hence, training is essential to bolster their confidence in conducting the intervention.	“I think that in general, the teachers’ community needs support… because there are many opinions expressed from popular knowledge, that is, from knowledge from their communities of origin, family, etc., but to say something like a contribution with serious knowledge I think we need… many times teachers are asked for many things… but teachers… do not have the tools, and they are being asked to comply with something without having the tools. So, I think it is important to provide them with those tools so that they can consciously apply them”(Teacher, co-educational high school) “I would like there to be more classes and more discussion” (Student, single-sex girls’ high school) “(It is necessary) to train teachers about the topics… because as I said, they can give orientation on several topics…, but the topic of sexuality is difficult for them” (Sex education program manager, single-sex girls’ high school)
Relevant topics for intervention	STI and pregnancy prevention methods Gender Relations LGBT Community Intimate Partner Violence Affectivity	Adolescents require scientific and up-to-date information to prevent STI and unwanted pregnancies Students and school professionals stress the importance of addressing sexism and gender discrimination Adolescents particularly emphasize the need for an intervention that is inclusive and does not adhere to heteronormative standards, but instead incorporates topics pertinent to LGBT adolescents. Adolescents and professionals highlight the need to include tools to prevent and address intimate partner violence. It should encompass a multidimensional perspective of sexuality, incorporating aspects of affectivity and mental health.	“Because I feel it is important, it is important to have biological and emotional sex education because if not, what will happen in the future? We won't know what diseases are transmitted, and some don't even know how to put on a condom; they don't know that they must press up… They pull down everything hard… it is clear evidence that we have a poor education respectively” (Student, single-sex boys’ high school) “I think it has to do with the appropriation of essential issues so that we can take it to the school environment. That is, it has to do with coexistence, that everyone has the floor, I don't know, that in a co-educational class, we don't give the floor only to the boys and not to the girls, that we take some to the blackboard and not others” (Teacher, single-sex girls’ high school) “I think that something that should be talked about is the topic of sexual orientations and genders. Because as they said before, it is not something that is talked about… so I think it would be important for it to exist, and for example, respect for, I don't know, pronouns, social names, and those things” (Student, single-sex girls’ high school) “Generally, more is said about sexuality on the heterosexual side, so there is a lack of information for people who are homosexual, lesbian or bisexual, or have a different orientation” (Student, co-educational high school) “There are still practices such as violence in the couple's relationship, which is an issue that we have to address now. There is still a lot of violence in the couple… as a relationship of domination, and the objectification of women, the “you are mine”, that happens a lot here” (Teacher, co-educational high school teacher)
Methodolo-gical considerations	Participato-ry strategies Interactive and fun activities	Students demand activities that enable them to take on leading roles within the intervention and cultivate their skills. Adolescents exhibit higher motivation when engaging in dynamic activities that offer enjoyment. This environment fosters learning and enhances adherence to the intervention.	“So the teacher made a way to make it more participatory, more dynamic, and then she started with the circles and questions that interested us” (Student, co-educational high school) “It depends on the type of class, because for example sometimes we were given guides… and you think that's… boring, and other classes that had questions and we stood up, those were more entertaining” (Student, co-educational high school)

#### 3.1.3 Final design of the intervention

Drawing on literature review insights and focus group/interview outcomes, the “Focus-on” Logic Model of Change was developed (see Supplementary Material 4). Although various personal and environmental determinants were identified in the Logic Model of the Problem, priorities were established in developing the Model of Change, focusing only on those considered most relevant by the literature and by the target population. For example, the key personal determinants selected for intervention efforts regarding risky sexual behavior include sexual health knowledge and risk perception.

Thus, specific objectives were developed in the Logic Model of Change aimed at addressing both personal and environmental determinants targeted by the intervention. It is important to note that these objectives were based on the selected theoretical framework, with the main foundation for “Focus-on” being the Theory of Planned Behavior (100).

This theory suggests that the intention to perform a behavior predicts subsequent behavioral change and that this intention comprises three cognitive variables (attitudes, subjective norms, and perceived behavioral control) (102). Additionally, this intention is best assessed through short-term self-reporting (immediate post-intervention assessment), while behavior change is generally measured in the medium or long term (follow-up assessment) (100, 103). Therefore, the objectives and outcomes developed in the Logic Model of Change are centered on skills, behavior and behavioral intention. The preliminary evaluation is specifically geared towards assessing intention.

On the other hand, components addressing the environmental determinants of this model encompass relational facets of sexuality such as the mitigation of discriminatory behaviors. These aspects were determined to be addressed with adolescents during workshop sessions and with facilitators through training.

**Figure 2:**
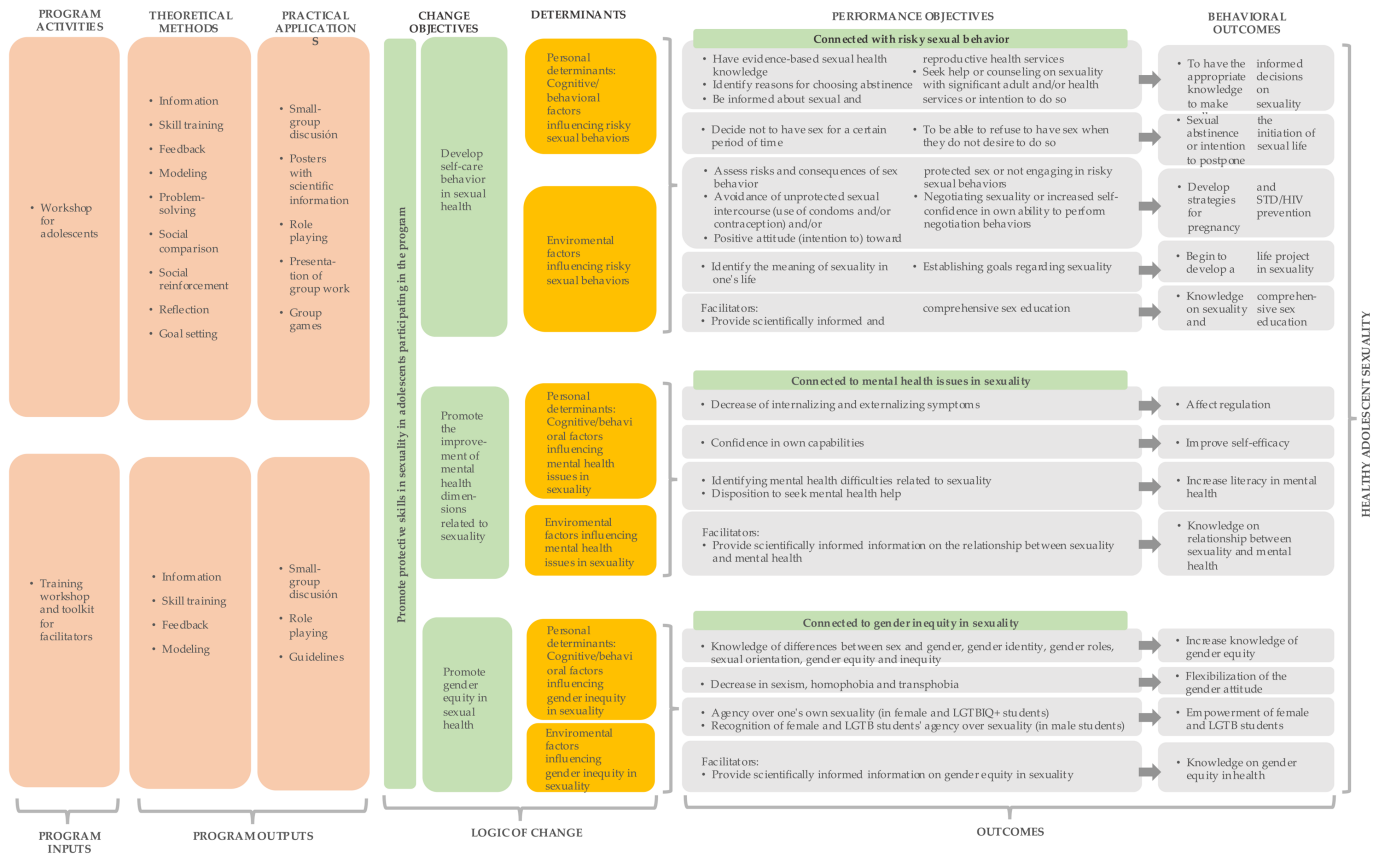
Intervention logic model (85).

Ultimately, through the synthesis of the problem definition (Problem Model) and the objectives for its modification (Change Model), the Intervention Logic Model was established (see Figure 2). Drawing from the findings of the systematic review, the intervention strategies most endorsed by the Theory of Planned Behavior (as well as other incorporated theories) were selected to achieve the objectives proposed in the Model of Change.

Thus, the Intervention Logic Model outlines an evidence-based, health equity-focused intervention structured around three pillars: self-care in sexual health, gender equity in sexuality, and mental health and sexuality. The program was named “Focus-on” (“Enfócate”, in Spanish) and includes:

• Facilitator training, which included the following topics: comprehensive approach to sex education, utilization of participatory methodologies in sexual education, adolescent mental health and sexuality, theory of emotion regulation, gender-sensitive perspective, Framework for Gender Transformative Health Promotion.
• Facilitator toolkit.
• High school workshops comprising 10 sessions with participatory activities, tailored to addresses identified needs collected through focus groups and interviews.

Theoretical foundations include the Theory of Planned Behavior (100), the FGTHP, and the Ecological Theory (104), supplemented by principles from the Social Cognitive Theory (105, 106), the Gross Model of Emotion Regulation (106), and the Transtheoretical Model of Change (107).

It's important to note that “Focus-on” can be integrated into preventive interventions targeting adolescents concerning various forms of risky behaviors. Given its comprehensive approach sexuality, the program can address intersections with other interventions (e.g., drug and alcohol use, school violence, sexual abuse) within the framework of its thematic pillars. Thus, a substance abuse prevention program could contribute to sessions under the “Mental Health and Sexuality” pillar, making it possible to address this risky behavior as a determinant for sexual health while simultaneously fostering skills to prevent its occurrence among adolescents.

### 3.2 Phase II: preliminary intervention validation and evaluation

#### 3.2.1 Validation of the intervention

Activities scoring below 70% agreement among the judges underwent modifications specific to the dimension that fell below this threshold (see Supplementary Material 4 for detailed results). These adjustments were aimed at enhancing the participatory nature of the activities, providing clear instructions to facilitate increased understanding among facilitators, and refining concepts to improve participant comprehension and encourage deeper reflections. Consequently, the final design of the sessions was refined, resulting in the completion of the sessions’ blueprint, which is outlined in Table 2 for review.

**Table 2:** “Focus-on” workshop sessions.

Session	Pillar	Activities	Theoretical basis	Topics
1: Focus on expressing yourself	Introduction to the program	Activity 1: Presentation of the program Activity 2: Our rules Activity 3: Teamwork Activity 4: The circle of sexuality	The transtheoretical model of change/Theory of planned behavior (subjective norms)	• Introduction to the program • Creation of norms of harmony • Expression and validation of beliefs, values and expectations
2: Focus on your experience	Self-care behavior in sexual health	Activity 1: Who do I ask?	Theory of planned behavior (subjective norms)/Theory of reflective learning	• Adolescence and sexuality (biological components, normative changes) • How and where to obtain adequate information (significant adults, health networks) • Reflection on the role of social media.
		Activity 2: Roads	Theory of planned behavior (attitude)/The transtheoretical model of change/Theory of reflective learning	
		Activity 3: Myths & Truths	Theory of planned behavior (subjective norms)/Theory of reflective learning	
3: Focus on getting informed	Self-care behavior in sexual health	Activity 1: I get informed to decide	Theory of planned behavior (subjective norms)/Theory of reflective learning	• Reproductive System • Pregnancy, STIs and HIV and their prevention
4: Focus on who you are	Gender equity in sexuality	Activity 1: Team Game	Framework for Gender Transformative Health Promotion/Theory of planned behavior (subjective norms)/Social learning theory	• Gender as a determinant of health • Sexual orientation and gender identity • Gender stereotypes and norms • Masculinities
		Activity 2: Feel different	Framework for Gender Transformative Health Promotion/Theory of planned behavior (attitude)/Theory of reflective learning	
5: Focus on who you are	Gender equity in sexuality	Activity 1: Expectations	Framework for Gender Transformative Health Promotion/Theory of planned behavior (subjective norms)/Theory of reflective learning	• Gender attitude (self-perception and interpersonal relations) • Masculinities • Equitable gender relations: empowerment and respect
		Activity 2: Putting Gender Equity into Practice	Framework for Gender Transformative Health Promotion/Theory of planned behavior (attitude, perceived behavioral control)/Theory of reflective learning	
6: Focus on your well-being I	Mental health and sexuality	Activity 1: The mental health pyramid	Social learning theory/Theory of reflective learning	• Mental health, affectivity, and sexuality • Identification of mental health states • Asking for help in mental health
		Activity 2: Mental health and sexuality	Theory of planned behavior (attitude, perceived behavioral control)/Theory of reflective learning	
7: Focus on your well-being II	Mental health and sexuality	Activity 1: True or False	Cognitive-behavioral theory/Theory of reflective learning	• Identification and regulation of emotions that influence sexual decision-making
		Activity 2: Influencers	Cognitive-behavioral theory/Theory of reflective learning	
		Activity 3: Shape your emotions	Theory of planned behavior (attitude, perceived behavioral control)/Cognitive-behavioral theory/Theory of reflective learning	
8: Focus on making decisions	Self-care behavior in sexual health	Activity 1: In your shoes	The transtheoretical model of change/Theory of planned behavior (subjective norms, attitude, perceived behavioral control)/Theory of reflective learning	• Sexuality decision-making (values-pleasure-affectivity-peer pressure) • Risky and protective sexual situations and behaviors (face-to-face and online) • Abstinence and safe sexual experiences
9: Focus on making decisions	Self-care behavior in sexual health	Activity 1: Practicing strategies	Theory of planned behavior (perceived behavioral control)/Social learning theory/Theory of reflective learning	• Self-care strategies in sexuality
10: Focus on your life project	Self-care/Mental health/Gender equity in sexuality	Activity 1: Balancing my decisions	The transtheoretical model of change/Theory of reflective learning	• Life project associated with sexuality • How I arrived and how I am leaving
		Activity 2: My plan	Framework for Gender Transformative Health Promotion/Theory of planned behavior (attitude, perceived behavioral control)/Theory of reflective learning	
		Activity 3: Closing the workshop	The transtheoretical model of change/Theory of reflective learning	

Subsequently, for step 4 of the IM, the program sequence was outlined: facilitator training, toolkit distribution, and workshop implementation for students. The intervention toolkit comprises three handbooks: (i) “Guide 1: Workshop Implementation,” detailing each session, (ii) “Guide 2: Focus-on Background,” offering theoretical insights into workshop topics. (iii) “Guide 3: Sex Education Methodology for Focus-on,” providing facilitators with methodological tools for conducting workshops with a participatory approach.

The toolkit content and graphic design prioritize cultural sensitivity and gender equity. For example, “Guide 1” incorporates relevant concepts and scenarios for the target population, while all guides use imagery free of gender stereotypes. “Guide 2” explores topics like “Gender and Adolescent Sexuality,” while “Guide 3” includes guidelines for culturally sensitive facilitation and inclusive language regarding gender and disability.

#### 3.2.2 Preliminary evaluation of focus-on

The reliability analysis of the ad-hoc survey initially employed the McDonald Omega coefficient to assess internal consistency. The coefficient yielded a value of *ω* = .8 for the total scale within the sample utilized for this study, indicating good reliability (94). This suggests that the instrument effectively evaluates protective skills in sexuality across its scale items.

Upon further examination, the sample distribution demonstrated non-normality, as evidenced by the Shapiro-Wilk test results (*W* = between .546 and .866; *p* = <.05). Consequently, the Wilcoxon-Mann-Whitney test was employed to assess changes in protective skills in sexuality among adolescents participating in the program.

The descriptive results indicate an increase in preventive skills related to sexuality among the participants. In Supplementary Material 6, the means of the pre- and post-intervention surveys by demographic characteristics can be observed. Additionally, the Wilcoxon-Mann-Whitney test results revealed statistically significant differences between pre and post-tests of this outcome (*W* = 58.5, *p* < .01), indicating an increase in its level following the intervention. For detail findings, please refer to Table 3 and Supplementary Material 7.

**Table 3:** Protective skills in sexuality pre- and post-intervention.

Outcome	*N*	Mean	Median	*SD*	*SE*
Protective skills in sexuality (pre)	30	25	25	4.94	.903
Protective skills in sexuality (post)	30	27	27	3.14	.573

A statistical analysis to identify statistically significant pre-post differences in protective skills in sexuality based on sexual orientation and gender identity could not be conducted due to the sample size. However, descriptive data before and after the intervention indicate that both participants identifying as cisgender and those identifying as non-cisgender showed an increase in their average score in protective skills in sexuality. The same trend was observed among heterosexual and non-heterosexual participants (this information can be viewed in Supplementary Material 8).

### 3.3 Next steps for “focus-on”

This study adhered to the IM guidelines for health promotion programs, completing up to step four of the process. Therefore, it's expected that future studies will address the remaining components of the next steps of IM. As such, the research team intends to formulate a plan for implementing and initially evaluating the program through a pilot randomized controlled trial. This pilot study aims to deliver the intervention on a small scale to assess various implementation aspects such as acceptability, feasibility, and preliminary results. These findings will inform refinements to design elements and procedural protocols for conducting a broader randomized controlled trial in the future, where the effectiveness of “Focus-on” and other implementation measures like adoption, adaptability, and fidelity will be assessed. This iterative process is recommended for the development of interventions across diverse health domains (108).

## 4 Discussion

Adolescence is a crucial period for sexual health, marked by intricate physical and psychological changes that can have profound impacts. Gender dynamics play a pivotal role in shaping adolescents' sexual behaviors and experiences (22, 109), and conventional gender norms embedded within sexual education interventions can perpetuate adverse outcomes, especially for LGBT adolescents (54, 55, 67, 68, 71). This highlights the importance of SEPs to address gender disparities and promote health equity, especially in low-income countries.

In response, this study aimed to design an SEPs tailored for adolescents, guided by the Intervention Mapping (IM) framework. Through intensive literature reviews, consultation interviews with educational professionals, and focus groups with high school students, we developed “Focus-on”- a comprehensive SEPs comprising facilitator training, a comprehensive toolkit, and a ten sessions workshop. Following the implementation of the program in two high schools with a cohort of 30 participants, as part of the fourth step in the IM process, we observed a statistically significant increase in protective skills in sexuality among students (*W* = 59, *p* = .01). Furthermore, based on the pre-post descriptive data, it was observed that -irrespective of sexual orientation or gender identity- all participants exhibited improvements in this domain. Thus, this intervention addresses a spectrum of factors influencing sex health outcomes and positively influencing facets such as pregnancy and STI prevention, which is promising for enhancing protective skills in sexuality.

These findings, first and foremost, underscore that the adoption of IM steps in the development of “Focus-on” facilitated the integration of the most robust evidence available with the current needs of the target population. Consequently, an evidence-based program was developed, as a way to effectively address the recipients' needs while remaining culturally relevant, adapting to the contextual nuances of its implementation.

Furthermore, the mapping of personal and environmental determinants offers a holistic perspective of the intervention by considering both individual and contextual factors. This approach aligns with the multidimensional framework of sexuality embraced in the design of “Focus-on”, which enables the identification of environmental influences on risky sexual behavior and address broader dimensions of sexual health beyond the biological realm. Consequently, the program not only targets individual factors but also equips adolescents to navigate challenges pertaining to sexuality within their environment. Additionally, while “Focus-on” includes a pillar dedicated to the biological aspect of sexuality (self-care), it incorporates two pillars aimed at fostering a comprehensive understanding of sexuality (mental health and gender).

Moreover, IM advocates for the integration of multiple theories in the design process, recognizing that synthesizing diverse theoretical perspectives facilitates and understanding of behavior change (98). This approach proved beneficial for the program's development, as while it predominantly draws from the Ajzen theory, it was augmented by additional frameworks essential for achieving the intervention's objective, such as the FGTHP framework, which enabled the gender-transformative approach of “Focus-on”.

Finally, the systematic nature of intervention planning with IM is noteworthy. Each step of this methodology encompasses specific components, participants, and outputs, contributing to an organized process in program development.

All these observations echo prior research findings from, further underscoring IM's efficacy in cultivating stakeholder consensus, tailoring interventions to contextual needs, and facilitating adoption and implementation processes (80, 110, 111).

Another noteworthy aspect revealed by this study is the gender-transformative approach of “Focus-on” and its comprehensive consideration of mental health dimensions associated with sexuality, thereby contributing to both global and local contexts. This contribution is particularly salient as many SEPs incorporate gender elements inconsistently due to insufficient understanding or implementation skills (46, 112), impeding their ability to generate changes in the gender-related areas that could impact sexual health outcomes. In this regard, “Focus-on” delineates specific objectives and sessions aimed at fostering positive attitudes and equitable gender relations in adolescent sexuality. Consequently, the program exhibits the potential to advance gender-equitable outcomes within adolescent sex education, as evidenced by the observed increase in protective skills in sexuality among participants representing diverse sexual orientations and gender identities. This situation can be linked to the inclusion of content and materials that embrace sexual diversity. Thus, the use of brochures and images (utilized in all sessions for group work) free from gender stereotypes, activities to challenge these stereotypes, and the training guides for facilitators (which promoted gender equity and its implementation in the workshop) were crucial elements. These principles have been highlighted in other studies as relevant for a gender-transformative approach (56, 59).

In this sense, it is possible to hypothesize that participants had an intervention experience that aligned with their needs, as it included the perspective of the target population in the design during the qualitative phase of the study. Moreover, by incorporating a comprehensive view of sexuality based on various sexual orientations and gender identities, all participants felt included in “Focus-on”, unlike gender-blind sex education programs that only benefit a specific group of adolescents (69, 70). These finding hold considerable significance, particularly in light of the potential shortcomings of SEPs lacking a gender-focused approach. Such inadequacies may diminish program effectiveness, exacerbate health issues, perpetuate gender stereotypes, evoke feelings of exclusion, and perpetuate stigma and discrimination (46, 113).

Similarly, the incorporation of mental health aspects within the intervention is noteworthy given their intrinsic connection to sexuality (8–12). Nonetheless, these dimensions receive comparatively less attention in sexual education initiatives (63), underscoring the need for programs capable of adequately addressing them.

Regarding strengths and limitations of this study, the main limitation pertains to the small sample size utilized for its preliminary evaluation, limiting the generalizability of results and precluding further statistical analyses regarding significant pre-post differences based on participants' sexual and gender orientations. Nonetheless, it's noteworthy that the sample size was deemed suitable for a preliminary evaluation. Conversely, a notable strength of the study lies in its innovative approach. The intervention adopts a comprehensive approach to sex education, with particular emphasis on addressing gender—a frequently- overlooked aspect in this type of SEPs (114). By leveraging the FGTHP framework, “Focus-on” goes beyond mere inclusion of gender-related content; it actively promotes gender equity in health, addressing issues like sexism, intimate partner violence, new masculinities, and discrimination against LGBT adolescents. Furthermore, employing IM to design the intervention ensures the development of an evidence-based, theoretically grounded program tailored to the specific needs of adolescents and intervention facilitators.

On the other hand, further examination of various aspects of “Focus-on” implementation, including its acceptability, feasibility, and subsequent effectiveness, is imperative. This comprehensive analysis will serve to broaden the generalizability of result. Additionally, it is necessary to persist in evaluating the intervention's role in promoting gender equity in health. Finally, it is imperative to sustain ongoing evaluation of the intervention's contribution to promoting gender equity in health.

## Data Availability

The raw data supporting the conclusions of this article will be made available by the authors, without undue reservation.
