# Supplementary material for: Promoting equity in adolescent health in Latin America: designing a comprehensive Sex education program using Intervention Mapping. A mixed methods study
Source: Front Reprod Health. 2024 Nov 18;6:1447016. doi: 10.3389/frph.2024.1447016 (PMC11609206; doi:10.3389/frph.2024.1447016)
Supplement: Supplementary file 1 [file Table1.docx]

**Supplementary Material 1**

Examples of the interview and focus group guide

**Examples of questions asked in interviews with school professionals**

1. How long have you been working with your high school's sex education program?

2. What has been your experience as part of this program?

3. Based on your experience and knowledge:

a) What are the needs and interests of students regarding sexuality?

b) What are the needs and interests of students regarding sex education?

c) What characteristics should a sex education intervention have to meet those needs and interests?

d) What types of activities should it include? (Please also consider the activities that have been most successful in your high school's sex education program.)

e) What topics should be addressed?

**Examples of questions asked in focus groups with students**

1. What comes to mind when you hear the words "sexuality" or "sexual health"?

2. What sex education activities have you participated in at your school?

3. What are your thoughts on those activities? (Positive aspects, areas for improvement)

4. Think about people your age:

a) What are the most significant challenges they face regarding sexuality?

b) Do you think sex education is needed to address those challenges? Why or why not?

5. What would motivate you to attend and stay engaged in a sex education intervention at school?

a) Types of activities

b) Topics covered

c) Other elements
