# Supplementary material for: Promoting equity in adolescent health in Latin America: designing a comprehensive Sex education program using Intervention Mapping. A mixed methods study
Source: Front Reprod Health. 2024 Nov 18;6:1447016. doi: 10.3389/frph.2024.1447016 (PMC11609206; doi:10.3389/frph.2024.1447016)
Supplement: Supplementary file 2 [file Table2.docx]

**Supplementary 2**

**Table.** Articles included for the development of the Logic Model of the Problem and Logic Model of Change

| **Author** | **Year** | **Article, book, book chapter, or report** |
| --- | --- | --- |
| Adimora, D. E., & Onwu, A. O. | 2019 | Socio-demographic factors of early sexual debut and depression among adolescents |
| Azevedo, JP., Favara, M., Haddock, SE., Lopez-Calva, LF., Müller, M., & Perova, E. | 2012 | Embarazo adolescente y oportunidades en América Latina y el Caribe. Sobre maternidad temprana, pobreza y logros económicos |
| Baltag, V., & Sawyer, S. M. (Eds.) | 2017 | International Handbook on Adolescent Health and Development: The public health response |
| Brawner, B. M., Jemmott, L. S., Wingood, G., Lozano, A. J., & Hanlon, A. L. | 2019 | Project GOLD: A pilot randomized controlled trial of a novel psychoeducational HIV/STI prevention intervention for heterosexually‐active black youth |
| Carvajal-Barona, R., Valencia-Oliveros, H. L., & Rodríguez-Amaya, R. M. | 2017 | Factores asociados al embarazo en adolescentes de 13 a 19 años en el municipio de Buenaventura, Colombia |
| CEPAL | 2019 | Primer informe regional sobre la implementación del Consenso de Montevideo sobre Población y Desarrollo |
| Chung, H. W., Kim, E. M., & Lee, J. | 2018 | Comprehensive understanding of risk and protective factors related to adolescent pregnancy in low‐ and middle‐income countries: A systematic review |
| Decker, M. J., Isquick, S., Tilley, L., Zhi, Q., Gutman, A., Luong, W., & Brindis, C. D. | 2018 | Neighborhoods matter. A systematic review of neighborhood characteristics and adolescent reproductive health outcomes |
| Díaz, D. I., Guerrero Parra, N. C., Robles Carreño, M. I., Rodríguez Medina, J., & Lafaurie Villamil, M. M. | 2020 | Hombres, salud sexual y salud reproductiva |
| Dides, C., & Fernández, C. (Eds.) | 2016 | Primer informe salud sexual, salud reproductiva y derechos humanos en Chile, estado de la situación 2016 |
| Doyle, C., Douglas, E., & O’Reilly, G. | 2021 | The outcomes of sexting for children and adolescents: A systematic review of the literature |
| Drewry, J., & Garcés-Palacio, I. C. | 2020 | Socio-Demographic Characteristics and Female Empowerment as Determinants of Adolescent Pregnancy in Colombia |
| Espinoza, M., Fernández, O. M., Riquelme, N., & Irarrázaval, M. | 2019 | La Identidad Transgénero en la Adolescencia Chilena: Experiencia Subjetiva del Proceso |
| Estrada, F., Suárez-López, L., Hubert, C., Allen-Leigh, B., Campero, L., & Cruz-Jimenez, L. | 2018 | Factors associated with pregnancy desire among adolescent women in five Latin American countries: a multilevel analysis |
| Fasula, A. M., Chia, V., Murray, C. C., Brittain, A., Tevendale, H., & Koumans, E. H. | 2019 | Socioecological risk factors associated with teen pregnancy or birth for young men: A scoping review |
| Finkelhor, D., Walsh, K., Jones, L., Mitchell, K., & Collier, A. | 2021 | Youth Internet Safety Education: Aligning Programs With the Evidence Base |
| Ganapathee, S., & Chandra Mouli, V. | 2021 | All Aboard the SRHR Train: Meeting young people’s SRHR needs during the COVID-19 pandemic |
| Ganchimeg, T., Ota, E., Morisaki, N., Laopaiboon, M., Lumbiganon, P., Zhang, J., Yamdamsuren, B., Temmerman, M., Say, L., Tunçalp, Ö., Vogel, J., Souza, J., & Mori, R. | 2014 | Pregnancy and childbirth outcomes among adolescent mothers: a World Health Organization multicountry study |
| Heidmets, L., Samm, A., Sisask, M., Kõlves, K., Aasvee, K., & Värnik, A. | 2010 | Sexual Behavior, Depressive Feelings, and Suicidality Among Estonian School Children Aged 13 to 15 Years |
| Hernández-Bello, L., Hueso-Montoro, C., Gómez- Urquiza, JL., & Cogollo-Milanés, Z. | 2020 | Prevalencia y factores asociados a la ideación e intento de suicidio en adolescentes: Revisión sistemática |
| Hersh, A. R., Saavedra-Avendaño, B., Schiavon, R., & Darney, B. G. | 2019 | Sexuality Education During Adolescence and Use of Modern Contraception at First Sexual Intercourse Among Mexican Women |
| Hillier, L., Jones, T., Monagle, M., Overton, N., Gahan, L., Blackman, J., & Mitchell, A. | 2010 | Writing Themselves in 3 (WTi3). The third national study on the sexual health and wellbeing of same sex attracted and gender questioning young people |
| Hollinsaid, N. L., Weisz, J. R., Chorpita, B. F., Skov, H. E., the Research Network on Youth Mental Health, & Price, M. A. | 2020 | The effectiveness and acceptability of empirically supported treatments in gender minority youth across four randomized controlled trials |
| Kågesten, A., Gibbs, S., Blum, R. W., Moreau, C., Chandra-Mouli, V., Herbert, A., & Amin, A. | 2016 | Understanding Factors that Shape Gender Attitudes in Early Adolescence Globally: A Mixed-Methods Systematic Review |
| Kirby, D., Lepore, G., & Ryan, J. | 2005 | Executive Summary: Sexual risk and protective factors. Factors affecting teen sexual behavior, pregnancy, childbearing and sexually transmitted disease: What are important? Which can you change? |
| Lavanderos, S., Haase, J., Riquelme, C., Morales, A., & Martínez, A. | 2019 | Embarazo Adolescente en Chile: Una mirada a la desigualdad sociodemográfica comunal |
| Leal F., I., Molina G., T., Luttges D, C., González A., E., & Gonzalez A., D. | 2018 | Edad de inicio sexual y asociación a variables de salud sexual y violencia en la relación de pareja en adolescentes chilenos |
| Leaper, C., & Brown, C. S. | 2018 | Sexism in Childhood and Adolescence: Recent Trends and Advances in Research |
| Mori, C.; Temple, J.R.; Browne, D.; Madigan, S. | 2019 | Association of Sexting With Sexual Behaviors and Mental Health Among Adolescents |
