# Supplementary material for: Promoting equity in adolescent health in Latin America: designing a comprehensive Sex education program using Intervention Mapping. A mixed methods study
Source: Front Reprod Health. 2024 Nov 18;6:1447016. doi: 10.3389/frph.2024.1447016 (PMC11609206; doi:10.3389/frph.2024.1447016)
Supplement: Supplementary file 3 [file Table3.docx]

**Supplementary 5**

**Table.** Expert judgment: Percentage of agreement among judges (presence of the evaluated dimension)

| **Sessions** | **Activity 1** | | | | | **Activity 2** | | | | | | | | **Activity 3** | | | | | | | | | | **Activity 4** | | | | | | |  |
| --- | --- | --- | --- | --- | --- | --- | --- | --- | --- | --- | --- | --- | --- | --- | --- | --- | --- | --- | --- | --- | --- | --- | --- | --- | --- | --- | --- | --- | --- | --- | --- |
|  | Precision | Relevance | Comprehensiveness | Adequacy | Precision | | Relevance | | Comprehensiveness | | Adequacy | | Precision | | | Relevance | | Comprehensiveness | | Adequacy | | Precision | | Relevance | | Comprehensiveness | | Adequacy | |  |  |
| 1 | 100% | 100% | 100% | 100% | 83.3% | | | 100% | | 83.3% | | 83.3% | | | 100% | | 100% | | 67% | | 67% | | 100% | | 100% | | 83.3% | | 100% | | |
| 2 | 100% | 100% | 100% | 100% | 100% | | | 100% | | 100% | | 100% | | | 75% | | 75% | | 100% | | 100% | |  | |  | |  | |  | | |
| 3 | 100% | 100% | 100% | 100% |  | | |  | |  | |  | | |  | |  | |  | |  | |  | |  | |  | |  | | |
| 4 | 100% | 100% | 80% | 80% | 100% | | | 100% | | 80% | | 80% | | |  | |  | |  | |  | |  | |  | |  | |  | | |
| 5 | 100% | 100% | 100% | 100% | 100% | | | 100% | | 100% | | 100% | | |  | |  | |  | |  | |  | |  | |  | |  | | |
| 6 | 67% | 100% | 67% | 33% | 100% | | | 100% | | 100% | | 67% | | |  | |  | |  | |  | |  | |  | |  | |  | | |
| 7 | 100% | 100% | 67% | 67% | 100% | | | 100% | | 67% | | 67% | | | 100% | | 100% | | 67% | | 67% | |  | |  | |  | |  | | |
| 8 | 100% | 100% | 100% | 100% |  | | |  | |  | |  | | |  | |  | |  | |  | |  | |  | |  | |  | | |
| 9 | 100% | 100% | 100% | 100% |  | | |  | |  | |  | | |  | |  | |  | |  | |  | |  | |  | |  | | |
| 10 | 100% | 100% | 80% | 80% | 100% | | | 100% | | 80% | | 80% | | | 100% | | 100% | | 60% | | 80% | |  | |  | |  | |  | | |
