# Supplementary material for: Promoting equity in adolescent health in Latin America: designing a comprehensive Sex education program using Intervention Mapping. A mixed methods study
Source: Front Reprod Health. 2024 Nov 18;6:1447016. doi: 10.3389/frph.2024.1447016 (PMC11609206; doi:10.3389/frph.2024.1447016)
Supplement: Supplementary file 4 [file Table4.docx]

**Supplementary Material 6**

**Table.** Pre and post-intervention survey means by demographic characteristics

| **Protective skills in sexuality** | **Demographics** | | | | | | | |
| --- | --- | --- | --- | --- | --- | --- | --- | --- |
|  | Nationality | | Religious belief | | | Age (years) | | |
|  | Chilean | Foreign | With religious belief | Without religious belief | Not sure | 14 | 15 | 16 |
| N | 20 | 20 | 9 | 16 | 5 | 10 | 12 | 8 |
| Mean |  |  |  |  |  |  |  |  |
| Pre | 21.1 | 22.2 | 20.4 | 21.6 | 23 | 21.5 | 21.3 | 21.8 |
| Post | 23.6 | 23.7 | 22.8 | 24.3 | 23.4 | 23.7 | 23.4 | 24 |
