# Supplementary material for: Promoting equity in adolescent health in Latin America: designing a comprehensive Sex education program using Intervention Mapping. A mixed methods study
Source: Front Reprod Health. 2024 Nov 18;6:1447016. doi: 10.3389/frph.2024.1447016 (PMC11609206; doi:10.3389/frph.2024.1447016)
Supplement: Supplementary file 5 [file Table5.docx]

**Supplementary Material 7**

**Table.** Pre and post-intervention survey descriptive statistics by item

| **Item** | **N** | | **Mean** | | **Median** | | ***SD*** | | **Min** | | **Max** | |
| --- | --- | --- | --- | --- | --- | --- | --- | --- | --- | --- | --- | --- |
|  | Pre | Post | Pre | Post | Pre | Post | Pre | Post | Pre | Post | Pre | Post |
| If I have sex, I use  Condom | 30 | 30 | 3.6 | 3.63 | 4 | 4 | .77 | .615 | 1 | 2 | 4 | 4 |
| I feel comfortable  with myself | 30 | 30 | 2.6 | 3.13 | 2.5 | 3 | 1.07 | .973 | 1 | 1 | 4 | 4 |
| I know where I must ask for appropriate  information about  sexual health | 30 | 30 | 3.13 | 3.47 | 3.5 | 4 | 1.01 | .730 | 1 | 2 | 4 | 4 |
| I know where I can go to get  medical attention in sexual health | 30 | 30 | 2.97 | 3.33 | 3.5 | 4 | 1.22 | .802 | 1 | 2 | 4 | 4 |
| I have enough (and appropriate)  information about  sexual health | 30 | 30 | 2.9 | 3.4 | 3 | 4 | 1.03 | .770 | 1 | 2 | 4 | 4 |
| I know when I need to ask for help when faced with difficulties in my mental health | 30 | 30 | 2.67 | 3.17 | 3 | 3 | 1.12 | .791 | 1 | 1 | 4 | 4 |
| I make the decisions about my sexuality (instead of my parents, friends, or partner) | 30 | 30 | 3.6 | 3.53 | 4 | 4 | .814 | .776 | 1 | 1 | 4 | 4 |
