# Supplementary material for: Promoting equity in adolescent health in Latin America: designing a comprehensive Sex education program using Intervention Mapping. A mixed methods study
Source: Front Reprod Health. 2024 Nov 18;6:1447016. doi: 10.3389/frph.2024.1447016 (PMC11609206; doi:10.3389/frph.2024.1447016)
Supplement: Supplementary file 6 [file Table6.docx]

**Supplementary Material 8**

**Table.** Pre and post-intervention descriptive results by gender identity and sexual orientation of Focus-on participants

| **Gender identity** | **Protective skills in sexuality (pre)** | | **Protective skills in sexuality (post)** | |
| --- | --- | --- | --- | --- |
|  | N | Mean | N | Mean |
| Female | 15 | 20.7 | 15 | 23.7 |
| Male | 12 | 22.5 | 12 | 23.7 |
| Other (e.g., gender fluid, he-she) | 3 | 21.3 | 3 | 23.3 |
| **Sexual orientation** |  |  |  |  |
| Heterosexual | 20 | 22.1 | 20 | 23.7 |
| Undefined | 3 | 18.7 | 3 | 25.3 |
| Bisexual | 4 | 20.3 | 4 | 22.5 |
| Other (e.g., pansexual, aromantic) | 3 | 21 | 3 | 23.3 |
