# Supplementary figures and images for: Promoting equity in adolescent health in Latin America: designing a comprehensive Sex education program using Intervention Mapping. A mixed methods study
Source: Front Reprod Health. 2024 Nov 18;6:1447016. doi: 10.3389/frph.2024.1447016 (PMC11609206; doi:10.3389/frph.2024.1447016)

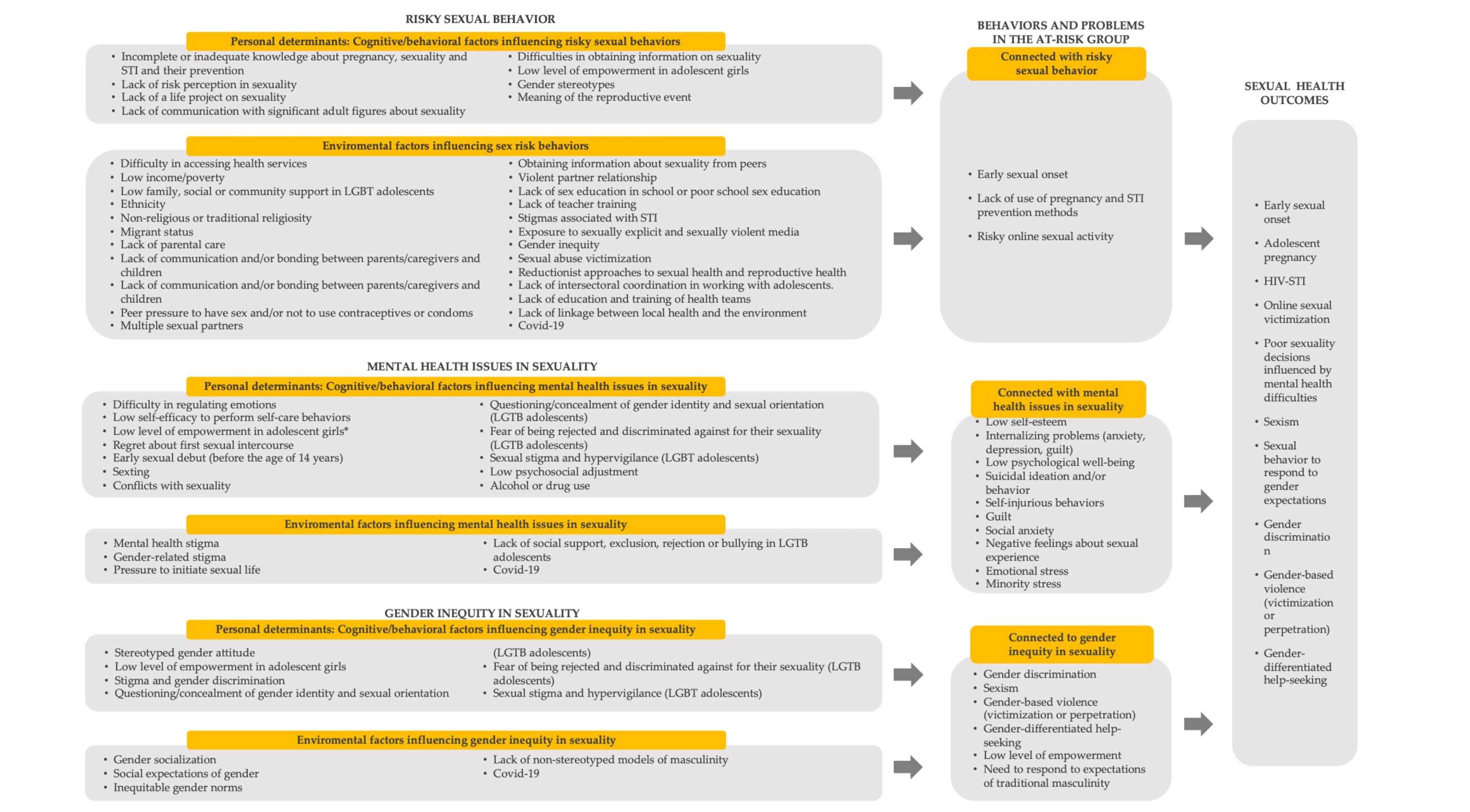

Supplement: Supplementary file 7 [file Image1.jpeg]

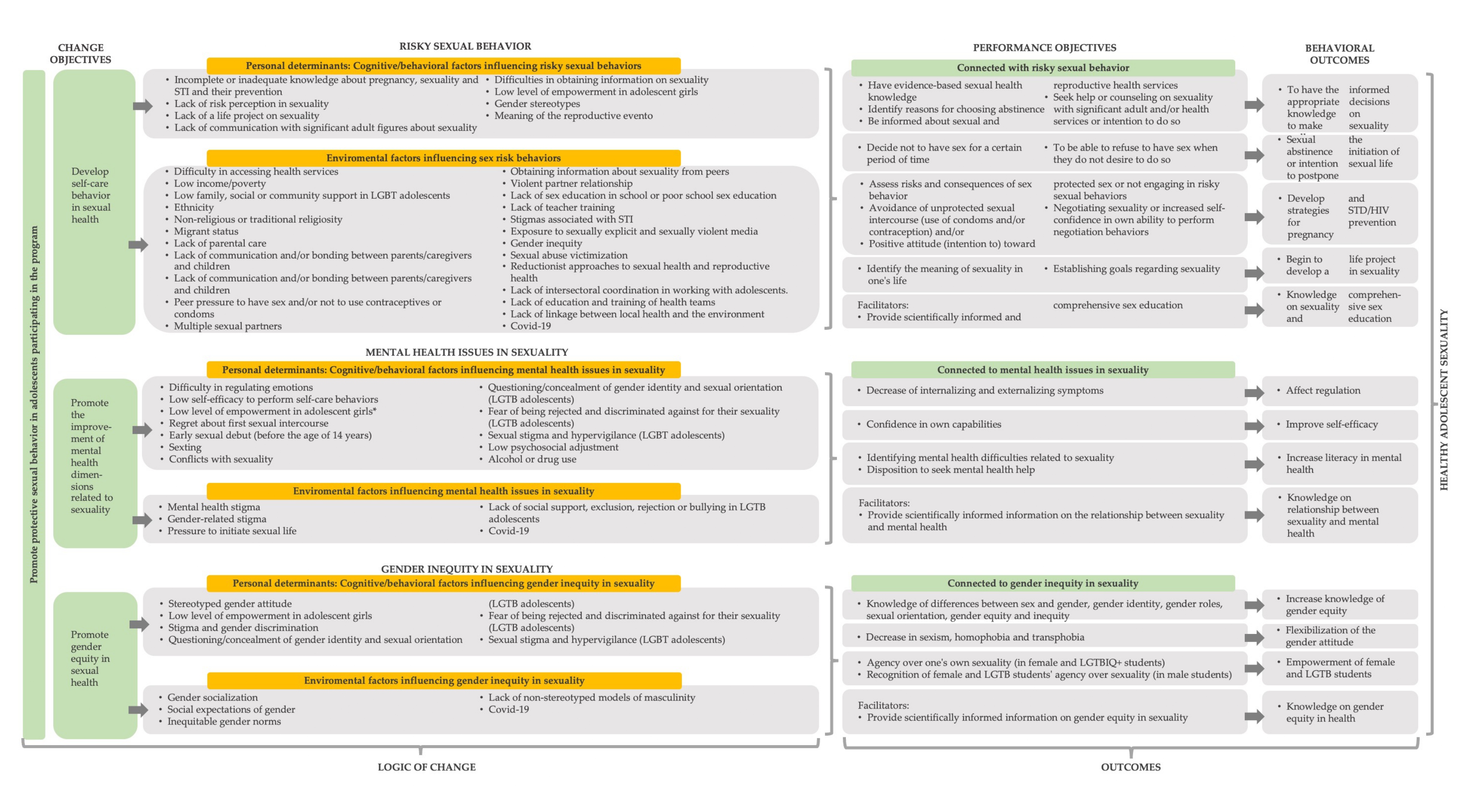

Supplement: Supplementary file 8 [file Image2.jpeg]
